# Supplementary figures and images for: Role of canonical and non-canonical cAMP sources in CRHR2α-dependent signaling
Source: PLoS One. 2024 Oct 2;19(10):e0310699. doi: 10.1371/journal.pone.0310699 (PMC11446442; doi:10.1371/journal.pone.0310699)

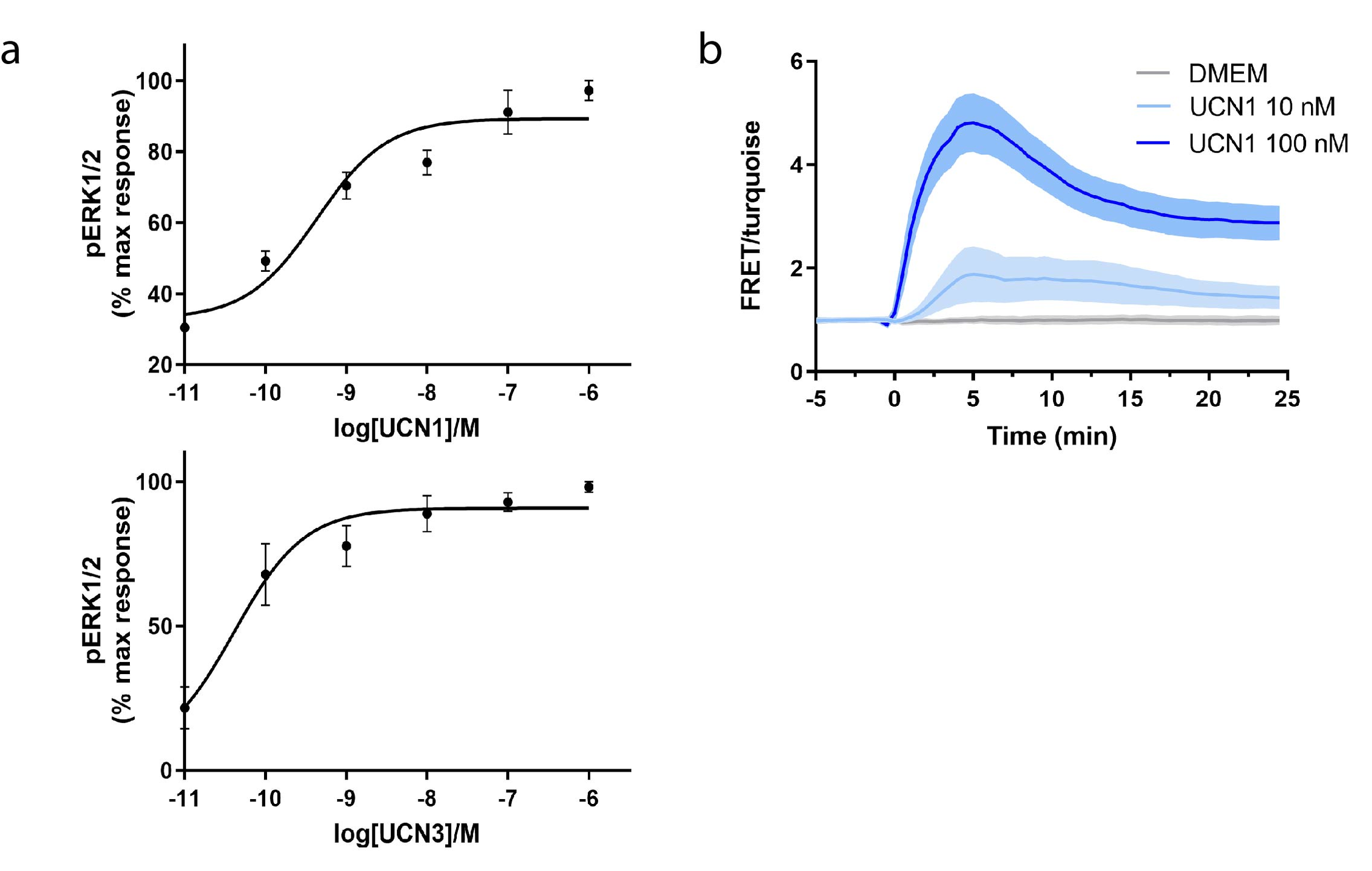

Supplement: S1 Fig — a, Cells were stimulated with UCN1 (upper) or UCN3 (bottom) at the indicated concentration for 6 minutes. pERK1/2 and total ERK1/2 were determined by western blot and quantified by densitometry analysis. pERK1/2 was normalized total ERK1/2 in each concentration point. Results are expressed as the percentage of maximum pERK1/2 obtained. Data: Mean ± SEM from 3 independent experiments. To determine EC50 for UCNs-induced ERK1/2 phosphorylation, data were adjusted with 3-parameter dose-response curve (EC50UCN1: 0,43 nM, EC50UCN3: 0,041 nM). b, Cells were transfected with FRET biosensors EPAC-SH187 stimulated with 10 and 100 nM UCN1. The curves illustrate the cAMP response over a 25-minute time frame. Data: Mean +/- SEM, n = 5–10 cells. (TIF) [file pone.0310699.s001.tif]

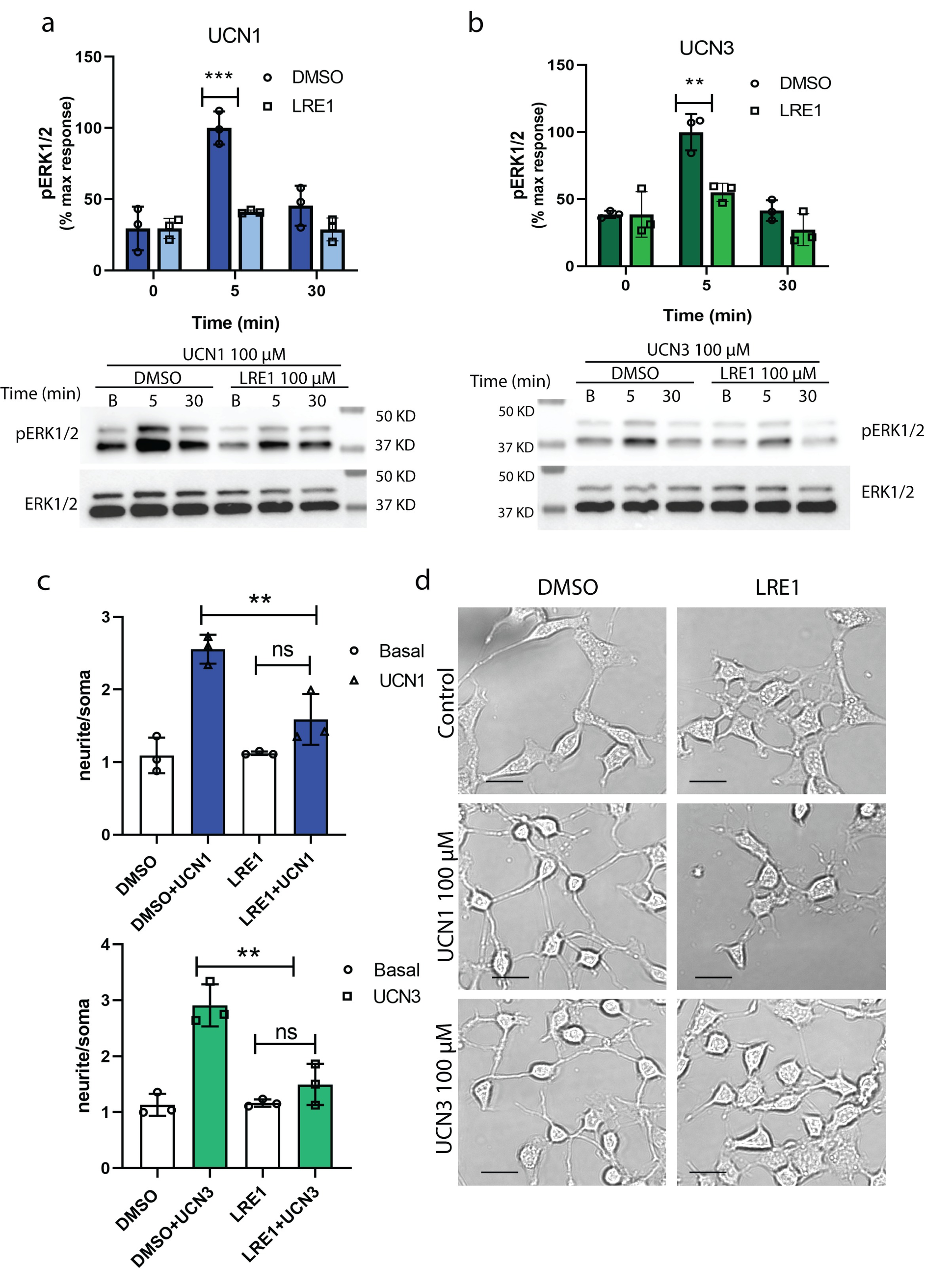

Supplement: S2 Fig — a-b, Cells were stimulated with 100 nM UCN1 (blue) or UCN3 (green) for the indicated time points in the presence or absence of 100 μM LRE1 specific sAC inhibitor. phosphorylated ERK1/2 and total ERK1/2 were measured by immunoblotting and quantified by densitometry using Fiji ImageJ software. pERK1/2 was normalized total to ERK1/2. Results are expressed as the percentage of maximum pERK1/2 obtained after stimulation. Data: Mean ± SEM, 3 independent experiments, **p<0,01, ***p<0,001 respect to basal by two ways ANOVA following by Tukey test. c-d, Cells were stimulated with 100 nM UCN1 (blue) or UCN3 (green) in the presence or absence of 100 μM LRE1 (sAC inhibitor). c, Neurite outgrowth was determined per cell after 15 min-pretreatment with inhibitors and 1h-treatment with the agonists, as the ratio between the longest neurite and the soma in each cell. Data: mean ± SEM, n = 3. ***, p<0,001 with respect to basal by repeated measures one-way ANOVA following by Tukey test. Bars: 20 μM. d, Representative photographs are shown for each treatment. (TIF) [file pone.0310699.s002.tif]

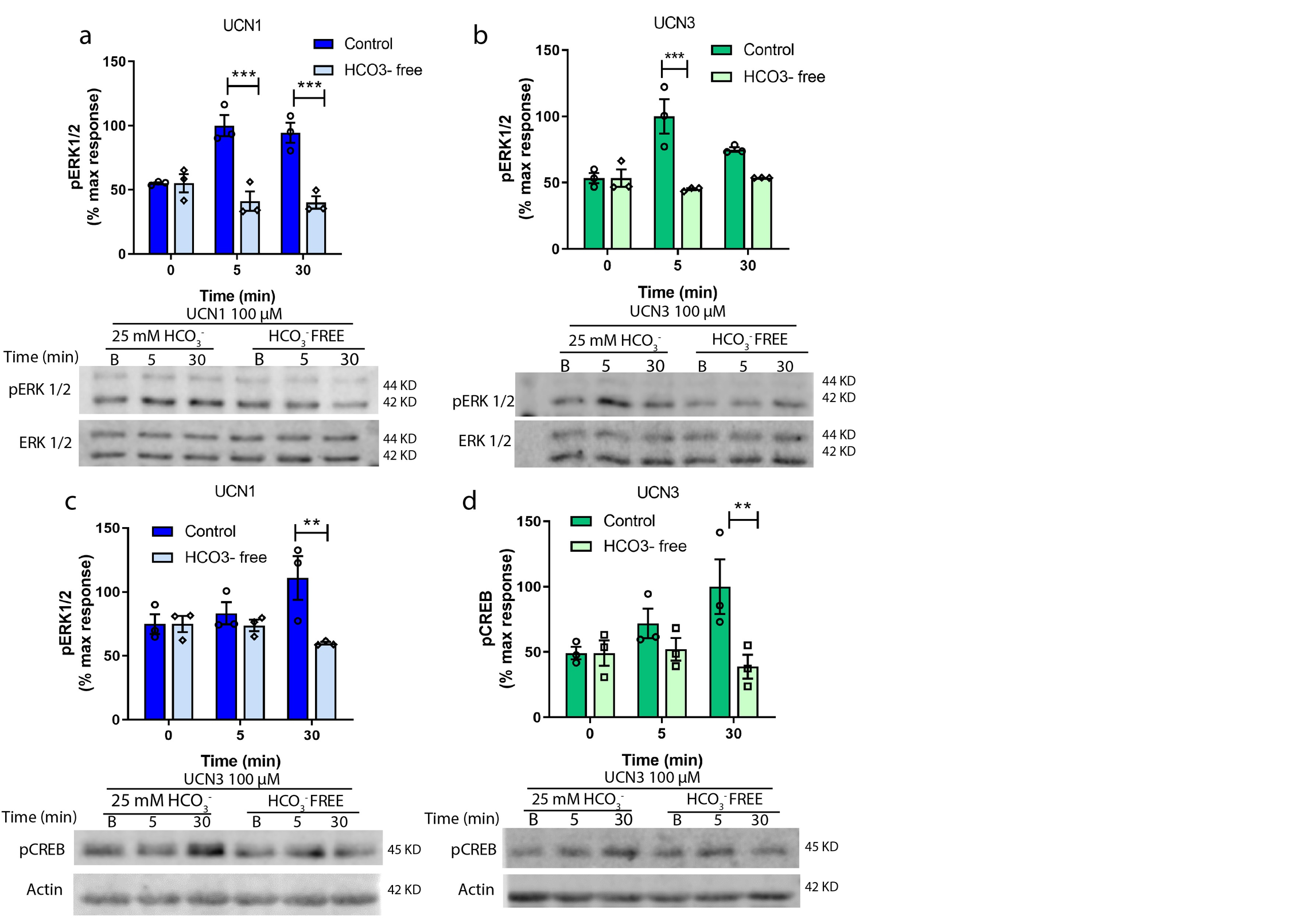

Supplement: S3 Fig — Cells were stimulated with 100 nM UCN1 (blue) or UCN3 (green) for the indicated time points in the presence or absence of bicarbonate (25 mM HCO3-) a-b, phosphorylated ERK1/2 and total ERK1/2 and c-d, phosphorylated CREB and Actin were measured by immunoblotting and quantified by densitometry using Fiji ImageJ software. pERK1/2 was normalized total to ERK1/2 and pCREB to Actin. Results are expressed as the percentage of maximum pERK1/2 or pCREB obtained after stimulation. Data: Mean ± SEM, 3 independent experiments, **p<0,01, ***p<0,001 respect to basal by two ways ANOVA following by Tukey test. (TIF) [file pone.0310699.s003.tif]

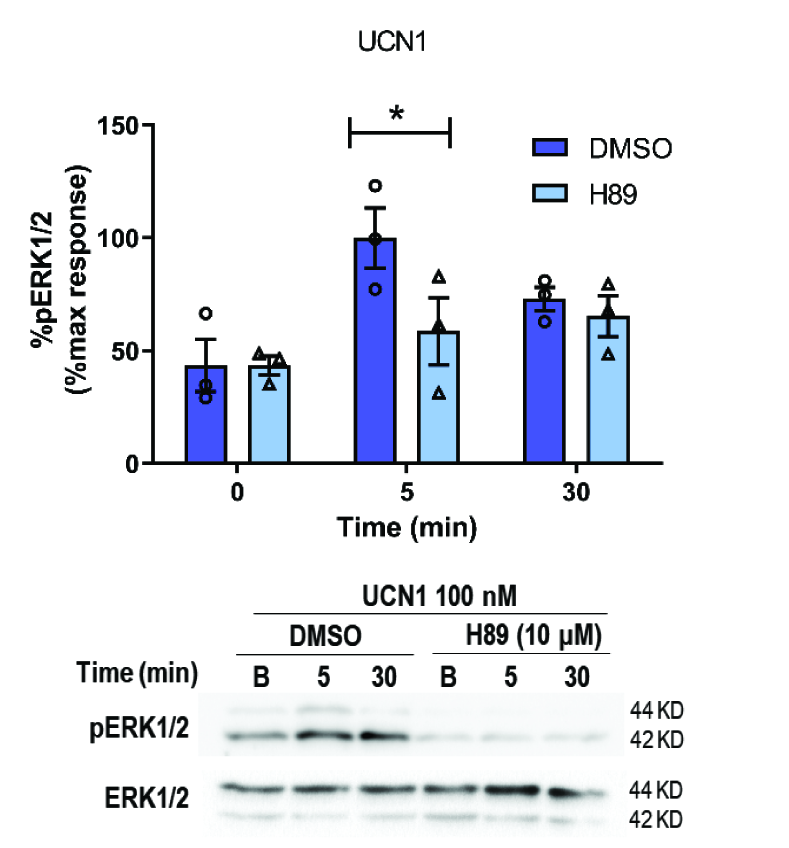

Supplement: S4 Fig — Cells were stimulated with 100 nM UCN1 for the indicated time points in the presence or absence of PKA activity inhibitor (10 μM H89), pERK and total ERK1/2 were measured by immunoblotting and quantified by densitometry using Fiji ImageJ software. pERK1/2 was normalized to total ERK. Results are expressed as the percentage of maximum pERK1/2 obtained after stimulation. Data: Mean ± SEM, 3 independent experiments, *p<0,05 respect with to basal by two ways ANOVA following by Tukey test. (TIF) [file pone.0310699.s004.tif]

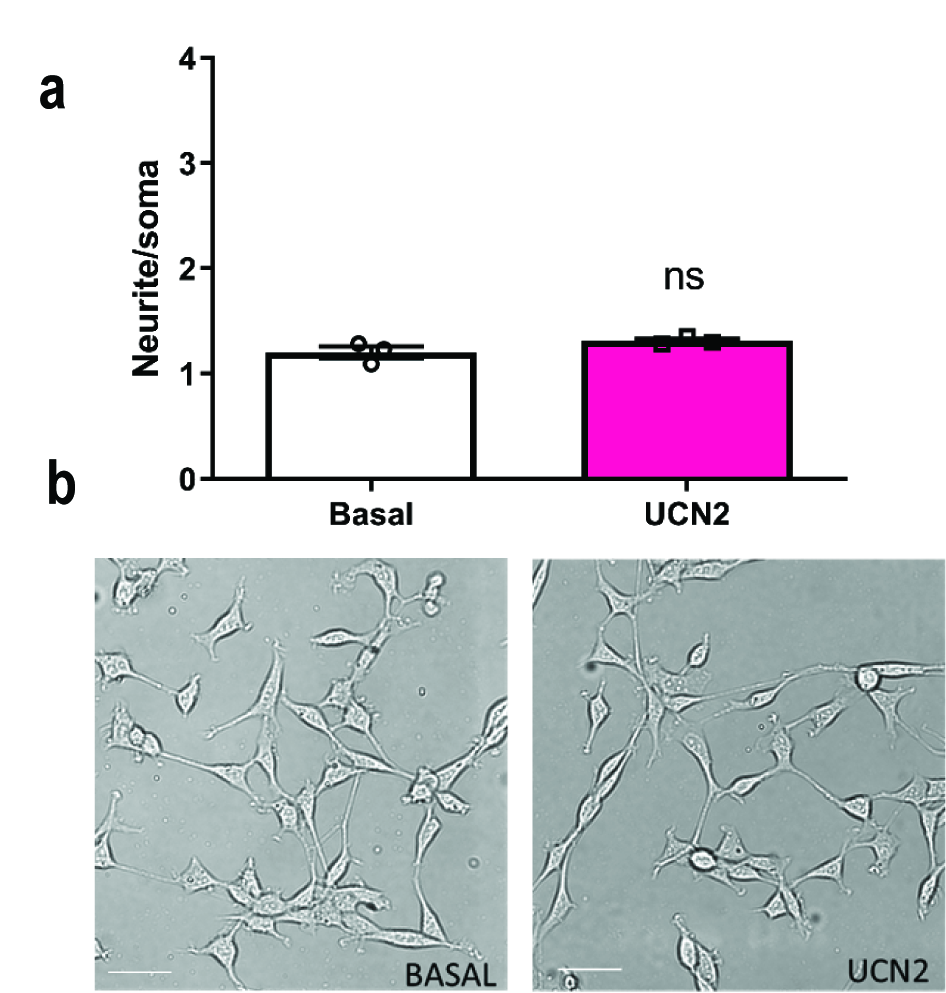

Supplement: S5 Fig — a, HT22-CRHR2α cells were stimulated with 100 nM UCN2 for 1h. Neurite outgrowth was determined in cells stimulated or in basal conditions. Data: Mean ± SEM 3 independent experiments, ns: no significative. b, Representative photographs are shown for the different treatments. Bars 50 μm. (TIF) [file pone.0310699.s005.tif]

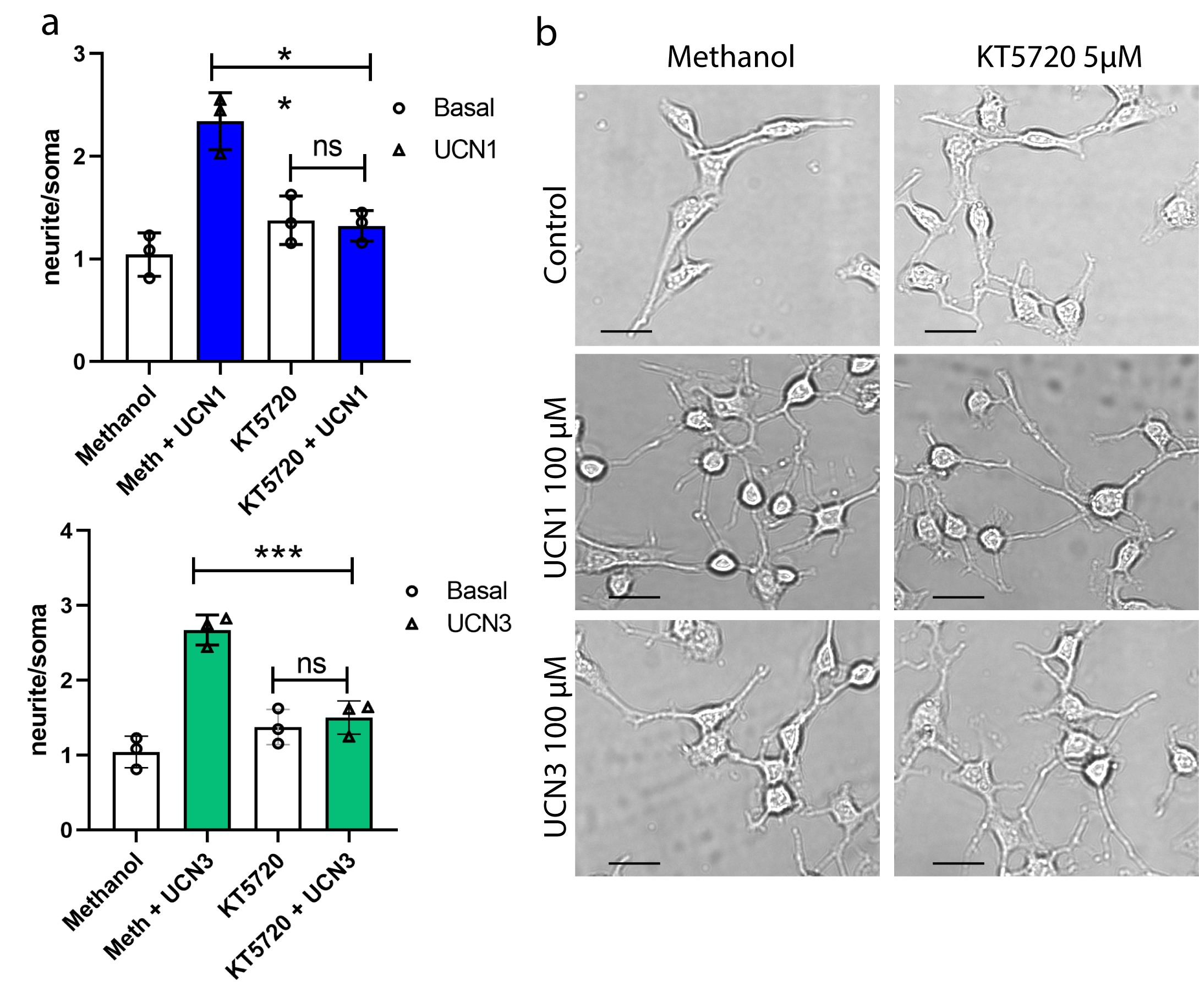

Supplement: S6 Fig — Cells were stimulated with 100 nM UCN1 (blue) or UCN3 (green) in presence or absence of PKA activity inhibitor (5 μM KT5720) a, Neurite outgrowth was determined per cell after 15 min-pretreatment with inhibitors and 1h-treatment with the agonists, as the ratio between the longest neurite and the soma in each cell. Data: mean ± SEM, n = 3. *, p<0,05 ***, p<0,001 ns: no significative with respect to basal by repeated measures one-way ANOVA following by Tukey test. b, Representative photographs are shown for each treatment. Bars 20 μM. (TIF) [file pone.0310699.s006.tif]

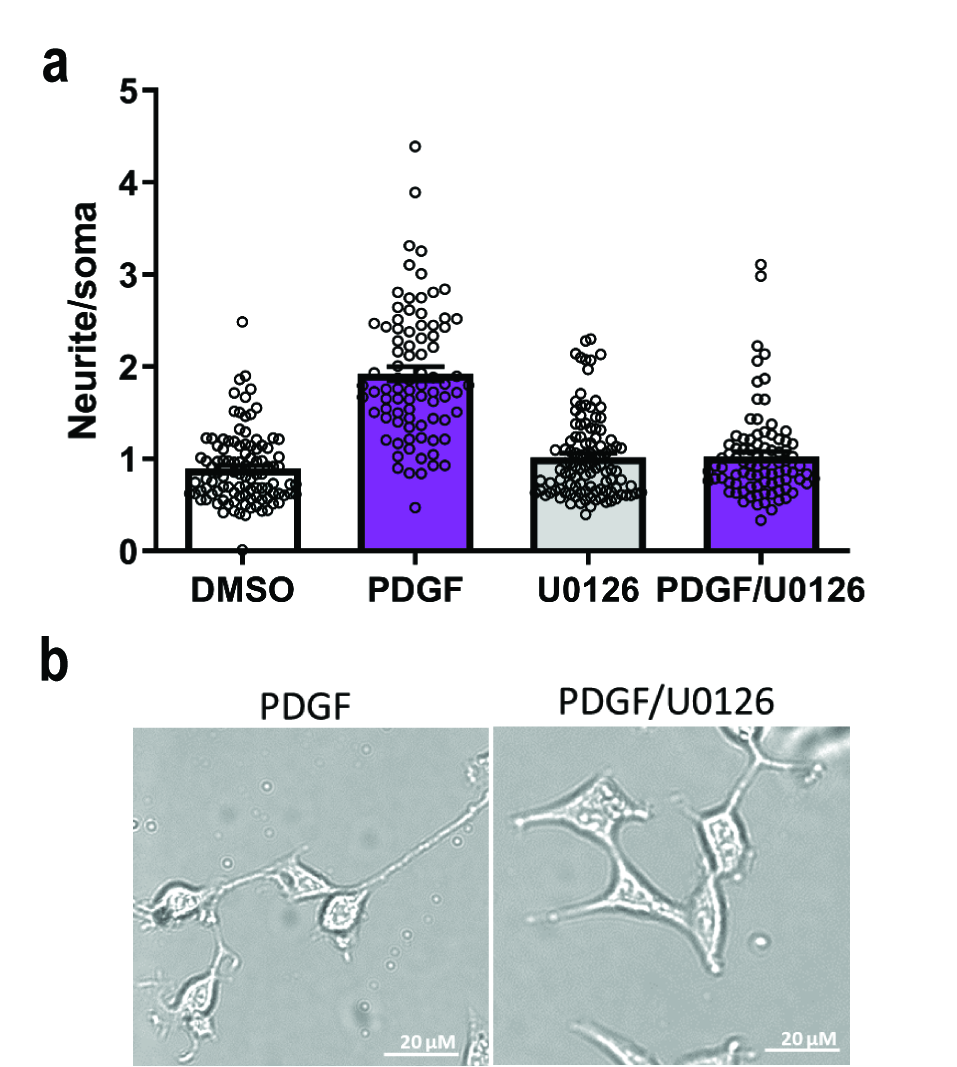

Supplement: S7 Fig — Cells were stimulated with PDGF (10 ng/ml) in the presence or absence of MEK inhibitor (10 μM U0126). a, Neurite outgrowth was determined per cell after 15 min-pretreatment with inhibitors and 1h-treatment with the agonists, as the ratio between the longest neurite and the soma in each cell. b, Representative photographs are shown for each treatment. Data: Mean ± SEM, n = 84–106 cells. (TIF) [file pone.0310699.s007.tif]

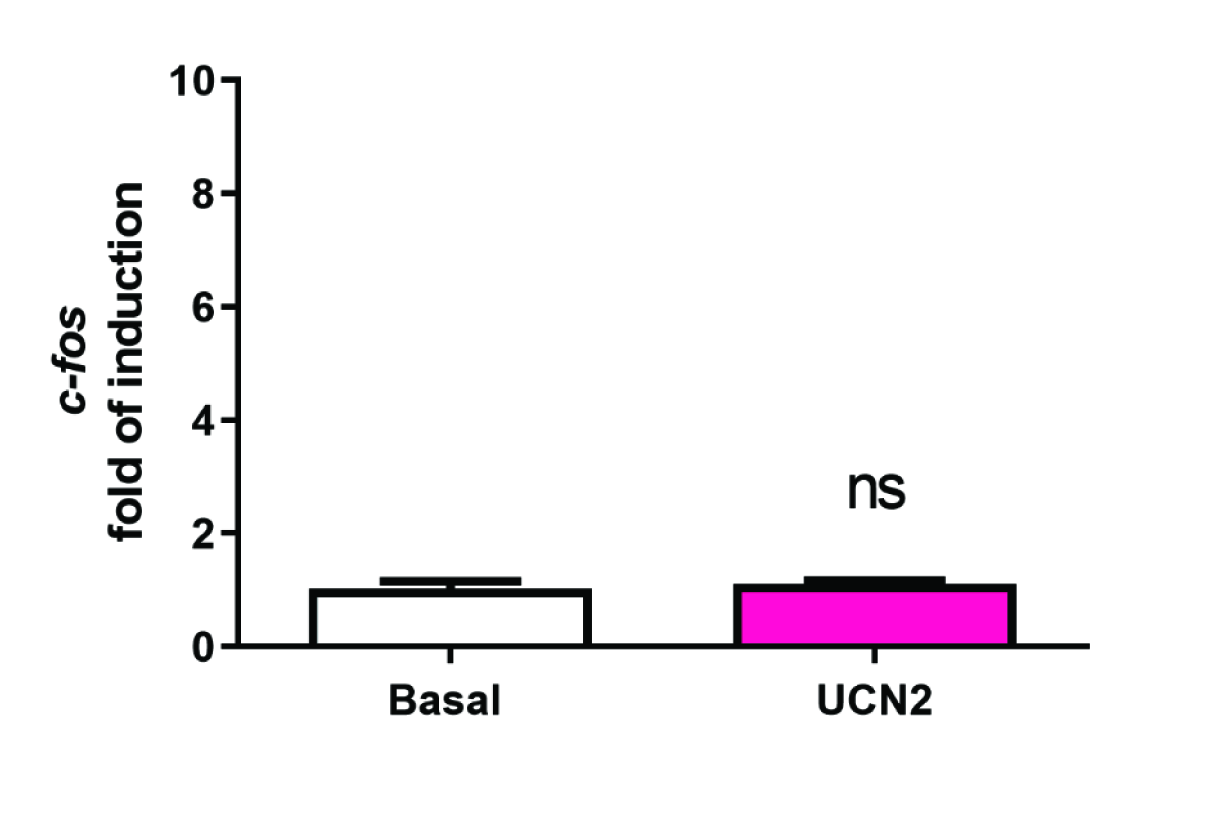

Supplement: S8 Fig — HT22-CRHR2α cells were stimulated with UCN2 100 nM ligands concentration for 1 hour. Transcription of c-Fos assesed by real time PCR normalized to HPRT is shown. Data: Mean ± SEM, 3 independent experiments, ns = no significative with respect to basal by two ways ANOVA following by Tukey test. (TIF) [file pone.0310699.s008.tif]
