# Supplementary material for: Role of canonical and non-canonical cAMP sources in CRHR2α-dependent signaling
Source: PLoS One. 2024 Oct 2;19(10):e0310699. doi: 10.1371/journal.pone.0310699 (PMC11446442; doi:10.1371/journal.pone.0310699)
Supplement: S1 Raw images — (PDF) [file pone.0310699.s009.pdf]

Blots Figure 1

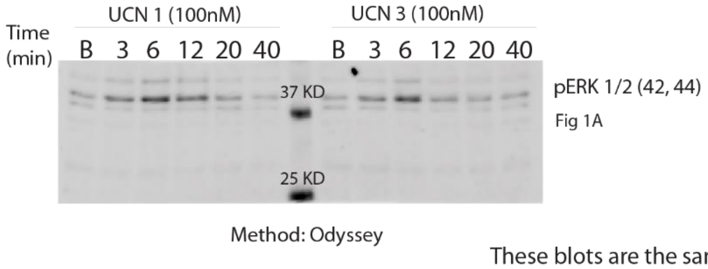

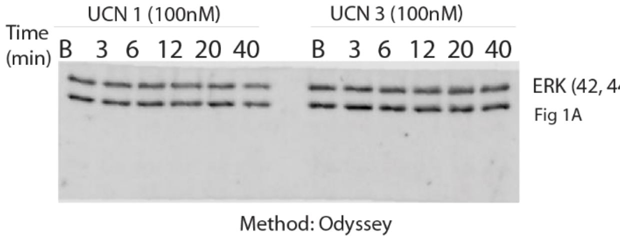

These blots are the same membrane revealed
with two different fluorophores

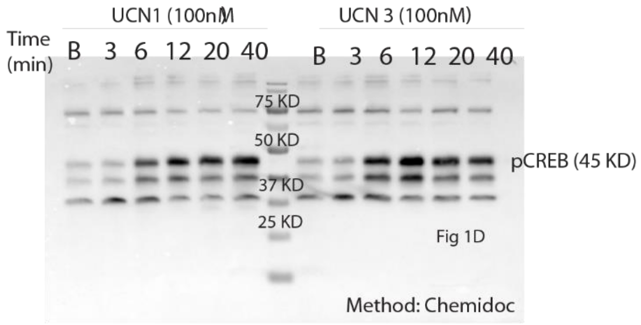

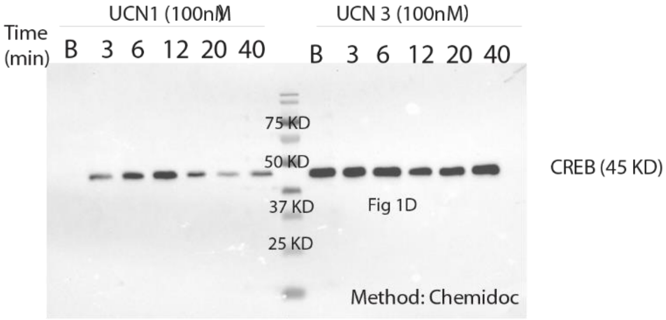

Blots Figure 2

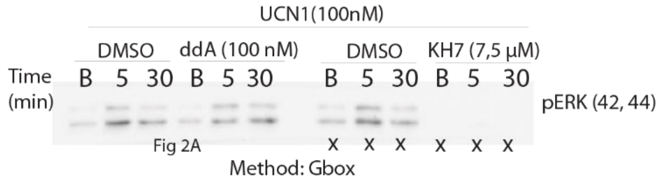

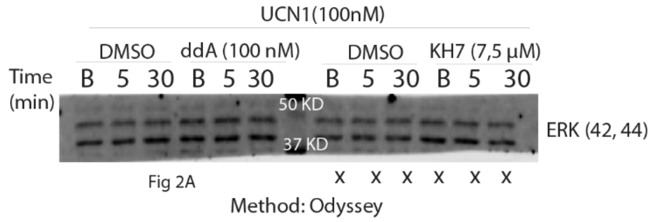

These blots are the same membrane revealed
with chemiluminiscence and fluorescence

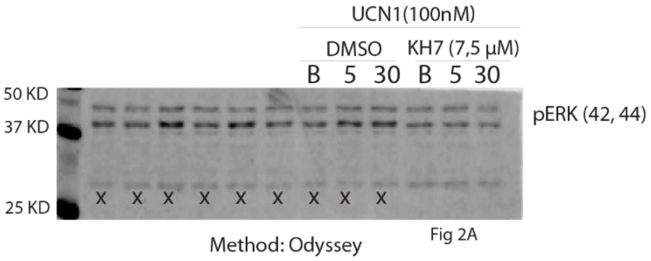

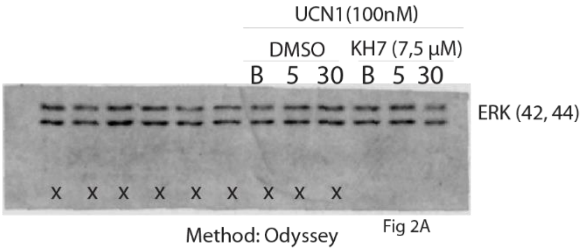

These blots are the same membrane revealed
with two different fluorophores

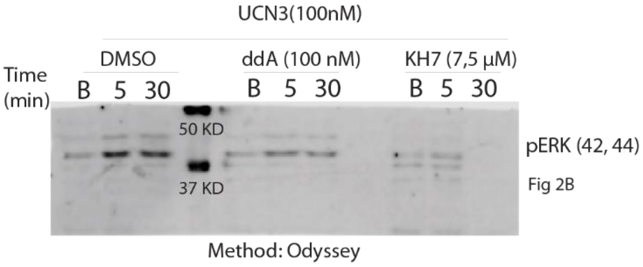

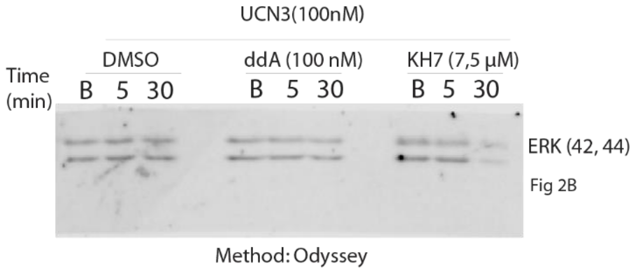

These blots are the same membrane revealed
with two different fluorophores

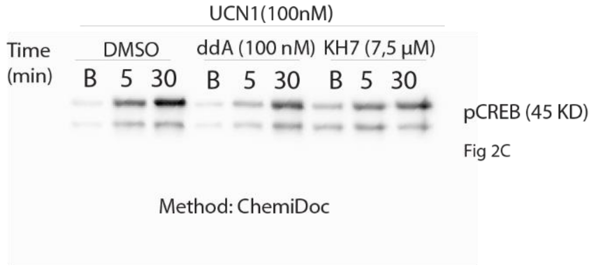

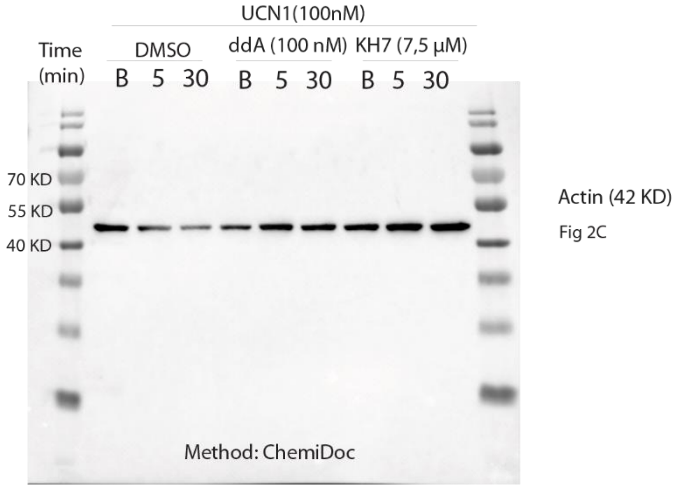

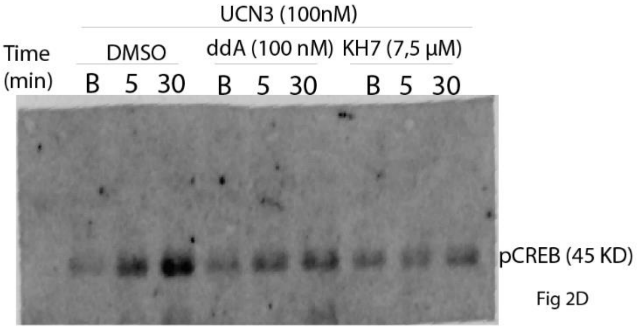

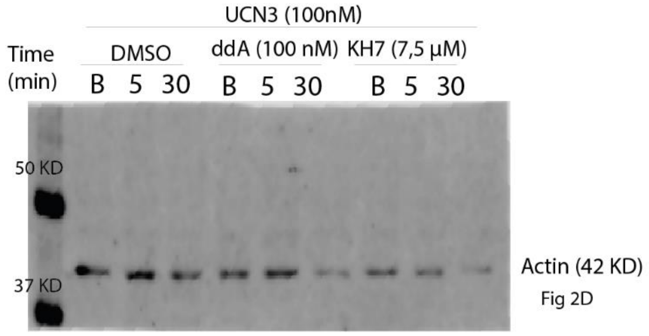

Method: Odyssey

Method: Odyssey

These blots are the same membrane revealed with two different fluorophores

Blots Figure 3

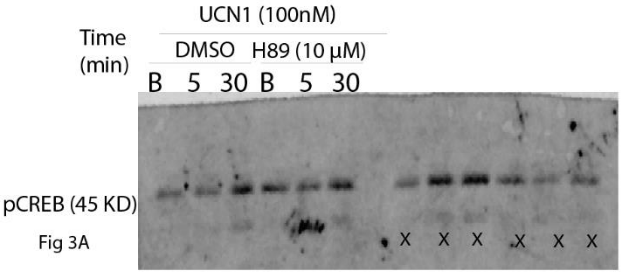

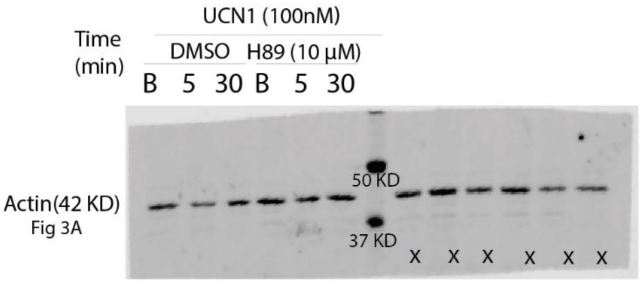

Method: Odyssey

Method: Odyssey

These blots are the same membrane revealed with two different fluorophores

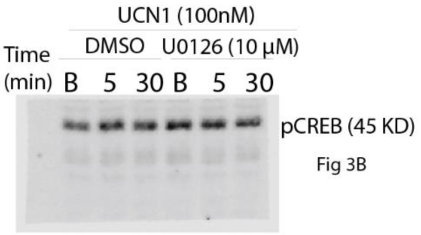

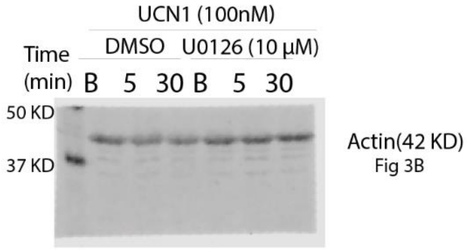

Method: Odyssey

Method: Odyssey

These blots are the same membrane revealed with two different fluorophores

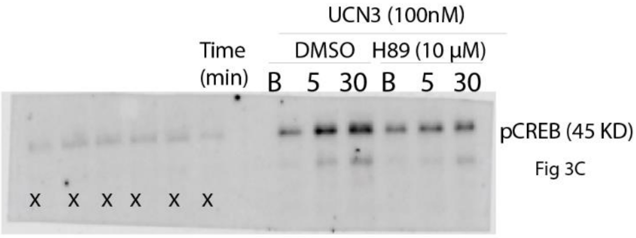

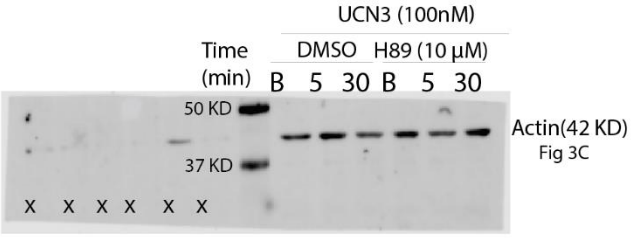

Method: Odyssey

Method: Odyssey

These blots are the same membrane revealed with two different fluorophores

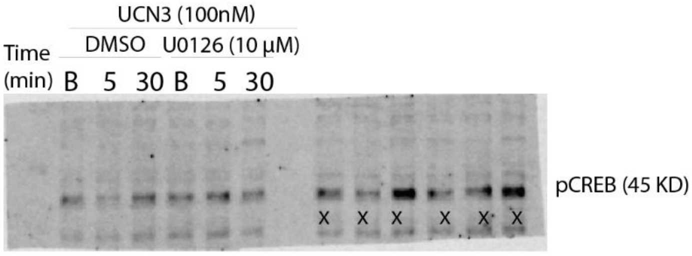

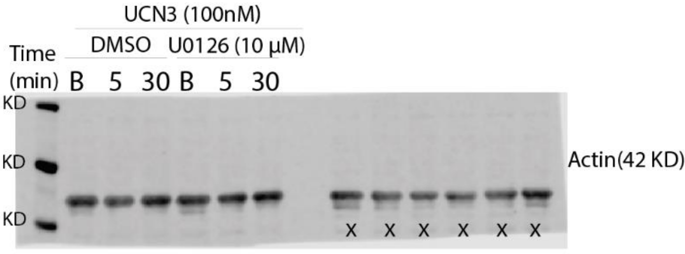

These blots are the same membrane revealed with two different fluorophores

Blots Supplementary Figure 3

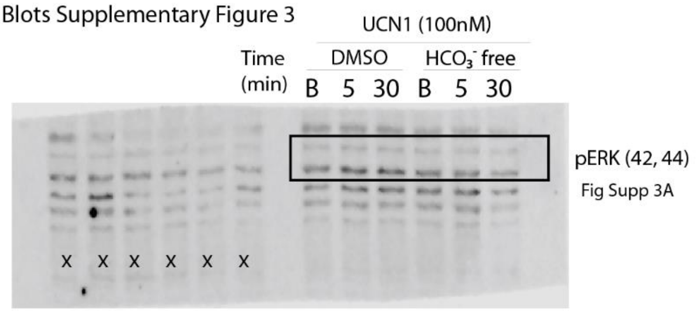

Method: Odyssey

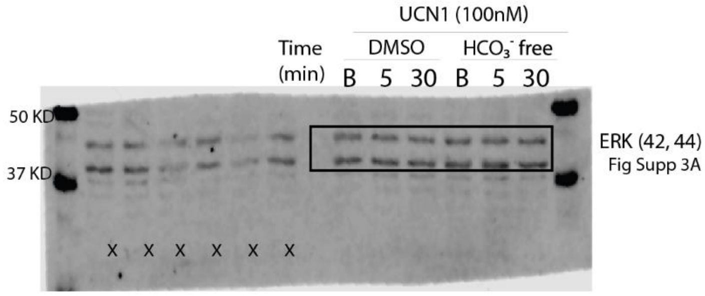

Method: Odyssey

These blots are the same membrane revealed with two different fluorophores

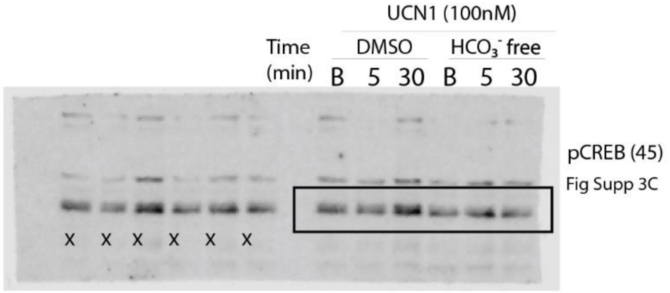

Method: Odyssey

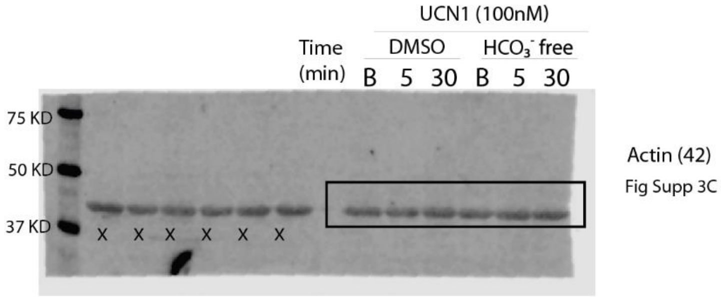

Method: Odyssey

These blots are the same membrane revealed with two different fluorophores

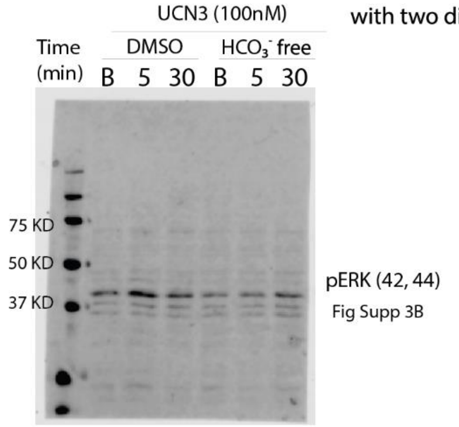

Method: Odyssey

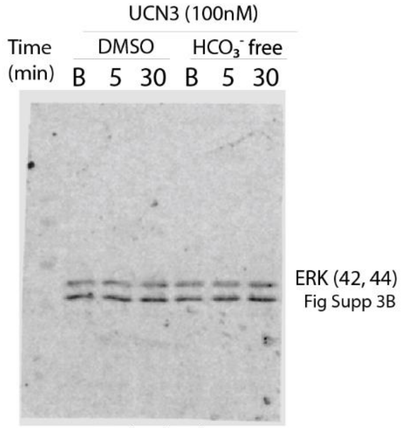

Method: Odyssey

These blots are the same membrane revealed with two different fluorophores

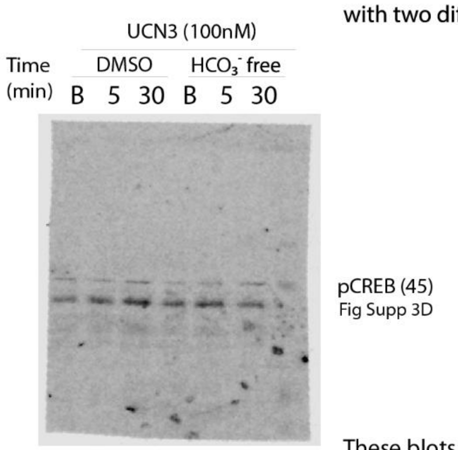

Method: Odyssey

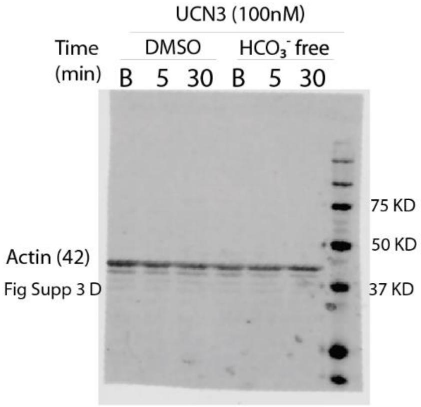

Method: Odyssey

These blots are the same membrane revealed with two different fluorophores

Blots Supplementary Figure 2

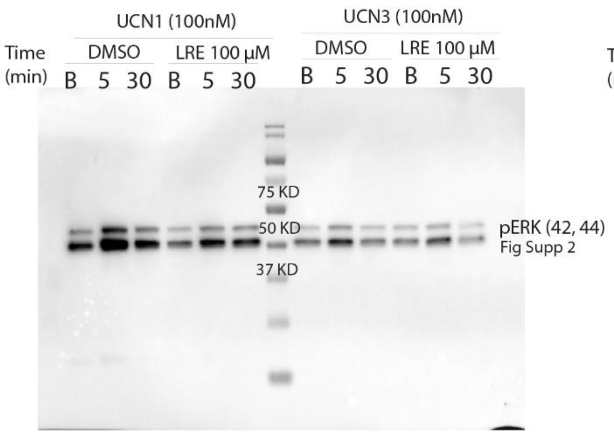

Method: Chemidoc

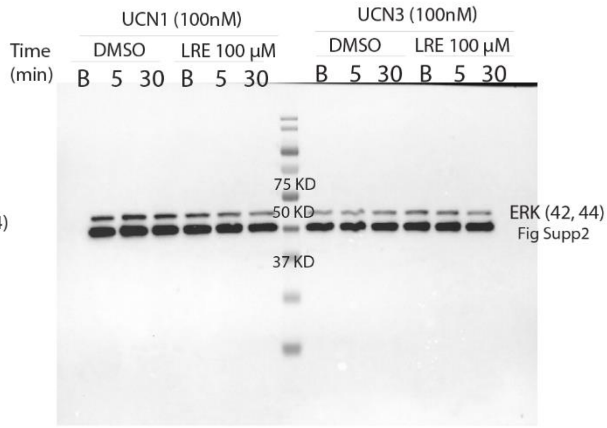

Method: Chemidoc

Blots Supplementary Figure 4

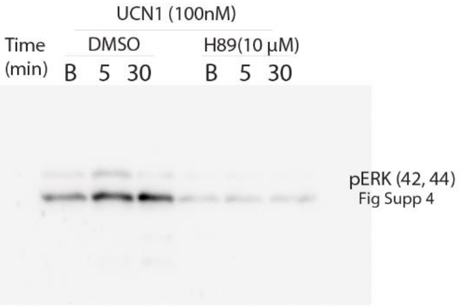

Method: Gbox

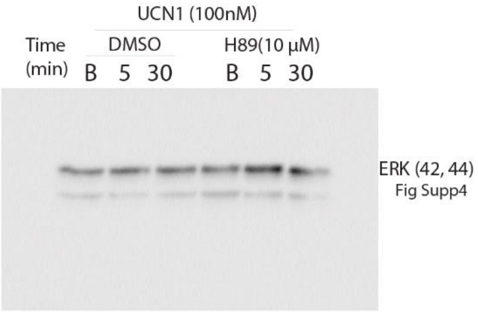

Method: Gbox
